# Pros and cons of mouse models for studying osteoarthritis

**DOI:** 10.1186/s40169-018-0215-4

**Published:** 2018-11-21

**Authors:** Santul Bapat, Daniel Hubbard, Akul Munjal, Monte Hunter, Sadanand Fulzele

**Affiliations:** 1 Department of Orthopedics Surgery, Augusta University, Augusta, GA 30904 USA; 2 Institute of Regenerative and Reparative Medicine, Augusta University, Augusta, GA USA

**Keywords:** Osteoarthritis, Animal models, Mouse, Horse, Guinea pig, Surgical, Chemically induced, Non-invasive

## Abstract

Osteoarthritis (OA) is one of the most common chronic conditions in the world today. It results in breakdown of cartilage in joints and causes the patient to experience intense pain and even disability. The pathophysiology of OA is not fully understood; therefore, there is currently no cure for OA. Many researchers are investigating the pathophysiology of the disease and attempting to develop methods to alleviate the symptoms or cure the OA entirely using animal models. Most studies on OA use animal models; this is necessary as the disease develops very slowly in humans and presents differently in each patient. This makes it difficult to effectively study the progression of osteoarthritis. Animal models can be spontaneous, in which OA naturally occurs in the animal. Genetic modifications can be used to make the mice more susceptible to developing OA. Osteoarthritis can also be induced via surgery, chemical injections, or non-invasive trauma. This review aims to describe animal models of inducing osteoarthritis with a focus on the models used on mice and their advantages and disadvantages that each model presents.

## Introduction

Osteoarthritis (OA) is one of the leading causes of disability in the United States. A total of 52.5 million American adults (22.7% of the adult population) report that their physician has diagnosed them with OA [1, 2]. Treatment costs for OA are also extremely high. Osteoarthritis of the knees alone is estimated to cost $185 billion per year [2]. Neither the current methods for diagnosing OA, nor the interventions used for preventing the progression of the disease are effective [3]. Currently, OA is diagnosed through physical examination and radiography. These methods are generally ineffective at definitively diagnosing the disease in its early stages, where interventions are more likely to be effective [4]. Current treatments include pain medications, weight loss, and surgical interventions. Corticosteroids are frequently used to reduce inflammation and alleviate pain. Surgical interventions including joint arthroplasty and joint replacement are also possible treatment strategies. These treatments have some efficacy in alleviating symptoms but do not stop the progress of the disease [3]. Even patients who undergo surgery for OA have a 10–20% chance of still experiencing symptoms post-surgery [3].

It is known that osteoarthritis is a disease that arises from wear and tear on the joints; in most cases it is a chronic disease but it can also arise as a result of trauma [3]. Over time, the wear and tear of normal activity leads to the progressive breakdown of cartilage and bone [3]. There is extensive research being conducted on the pathophysiology of OA as well as on therapies to alleviate the pain and to slow or even stop the progression of the disease. Human clinical studies are very difficult to conduct for the purpose of understanding the pathophysiology of disease development for several reasons. The disease presents differently in each patient which makes if difficult to make accurate conclusions about the progression of the disease [4]. Furthermore, the disease is chronic and takes years to develop in humans, which makes studies time consuming and expensive. Finally, there are serious ethical issues with using humans as test subjects as it is clearly unethical to induce OA in humans for studies. Studies on therapeutic agents must be conducted on animal subjects before they can be conducted on humans. These challenges serve as major impediments for studies of OA using human subjects [5]. Many studies on OA use animal models to study the progression of the disease and evaluate the efficacy of therapeutic methods. The advantages of animal models include rapid onset, the ability to control the severity of the disease, similar anatomy and disease progression to humans, and the ability to test and follow a large number of subjects ethically. Therapeutic agents in particular must be tested on animals before they can be administered to humans [5, 6]. Our aim is to summarize the different mouse models used to mimic OA for the purpose of studying the pathophysiology and progression of the disease as well as for testing possible therapeutic agents. Furthermore, we will examine the advantages and disadvantages of each of these models and determine their utility in studying the pathophysiology of OA and evaluating treatments. Other animal models will be addressed briefly along with the cases in which they might prove more effective than murine models.

### Importance of murine osteoarthritis models

Many different species of animals are used to mimic OA. Studies have been conducted on mice, rats, rabbits, guinea pigs, dogs, pigs, horses, and other animals. Most researchers studying OA in animal models use mice in their studies for several reasons. First and foremost, mice have a musculoskeletal system that develops quickly [7, 8]. This allows a large number of subjects to be raised and tested in a short period of time. Furthermore, mice are relatively cheap, which makes them an economical choice for studies which require a large sample size. The ability to raise a large number of subjects quickly and economically makes mice an ideal choice for studies examining the pathophysiology and progression of OA. Mice are also commonly used as the first line of testing for drugs and other therapies; however, differences in anatomy and physiology between humans and mice make mice an imperfect model for studying interventional methods [5, 6]. Therefore, this review will focus on murine models but briefly address other animal models to show when they might be more applicable. There are many different models that are used for mimicking OA in mice for studies. There are spontaneous models of OA, which include naturally occurring osteoarthritis and genetic modifications that cause the mice to be more likely to develop osteoarthritis. There are also surgically induced and chemically induced models of OA. Finally, there are non-invasive induced models of OA which use applied forces to cause trauma in the joint. This trauma causes injuries which eventually lead to the development of OA in the knee of the subject [7]. Each of these models will be described in greater detail in the next section along with their advantages and disadvantages. These advantages and disadvantages are also summarized in Table 1.

**Table 1:** This table summarizes the basis, time of onset, advantages and disadvantages of each of the OA models discussed in this paper

Model type	Model name and basis	Time of onset	Pros	Cons	References
Spontaneous	Naturally occurring—occurs as a result of wear and tear over the subject’s life	~ 4–6 months old	Most natural progression No specially trained personnel required No specialized equipment needed	Long progression time Low incidence High cost Variability in severity and onset	[5, 9, 10]
Spontaneous	Genetically modified—occurs as a result of natural wear and tear over the subject’s life with higher susceptibility due to genetic selection	~ 18 weeks old	Natural progression High incidence No specially trained personnel required Possibility of assessing impact of genetics in OA development and possible therapeutic targets	Low generalizability High cost Possibility of confounding symptoms as a result of genetic alterations Variability in severity and onset	[5, 10–22]
Surgically induced	ACL transection—joint instability	~ 3–10 weeks depending on method	High incidence Rapid progression Useful in assessing therapies	Need specially trained surgeon Risk of infection	[5, 6, 10, 24]
Surgically induced	Medical meniscectomy—joint instability	~ 4 weeks post-surgery	High incidence Rapid progression Useful in assessing therapies	Need surgeon Risk of infection	[5, 6, 10, 25–27]
Chemically induced	Mono-iodoacetate—joint inflammation and chondrotoxicity	~ 6 weeks from injection	High incidence Rapid progression Useful for studies in pain behavior	Progression not similar to natural progression in humans Only useful for studying pain behavior	[5, 10, 32, 33]
Chemically induced	Collagenase—joint inflammation and collagen breakdown	~ 3 weeks from injection	High incidence Useful for studies in pain behavior Useful for studies in potential therapies Most rapid progression	Progression not similar to natural progression in humans	[5, 10, 32, 33]
Non invasive induction	Intra-articular tibial plateau fracture—trauma to the knee and joint destabilization	~ 8–10 weeks post injury	Useful in replicating acute traumatic OA (i.e. from accidents) Rapid progression Severity of the lesions can be altered to compare effects of different Forces. Low risk of infection Repeatable	Need specialized equipment Not useful in modeling chronic onset OA	[5, 10, 40, 42, 43]
Non-invasive induction	Cyclic articular cartilage tibial compression—repeated trauma to the knee and joint destabilization	~ 8–10 weeks post injury	Highly effective for studying chronic overuse Injuries. Low risk of infection Repeatable	Need specialized equipment Lengthy induction Several cycles needed Not effective for studying acute PTOA	[5, 10, 40, 42, 43]
Non-invasive induction	Anterior cruciate ligament (ACL) rupture via tibial compression overload—single powerful trauma and joint destabilization	~ 8–16 weeks post injury	Single traumatic load is enough to induce OA Rapid progression Lower risk of infection Repeatable	Need specialized equipment	[5, 10, 50, 53]

## Spontaneous models

### Naturally occurring osteoarthritis

Spontaneous models are the simplest model for mimicking OA in mice. In spontaneous models of OA, the natural aging process and its effects on the subject animals’ joints are responsible for creating OA [9]. In these cases, the subject mice are not subjected to any treatment; many of these mice will still develop OA as a natural part of their aging process. The progression of OA in these mice closely mimics the progression of non-traumatic OA in humans as a result of natural wear and tear throughout the course of life [9]. These models are useful because the similarity between the model and osteoarthritis in humans enables researchers to observe and study a completely natural onset and progression of OA; researchers can also test various therapeutic methods on mice that have spontaneously occurring OA. This means that spontaneous models of OA are applicable to a wide variety of studies about OA [9]. Also, it is relatively easy to implement as there is no specialized equipment needed, nor is there a need for a surgeon with specific training to perform a procedure to induce OA [10]. However, there are certain drawbacks to spontaneous models. Osteoarthritis develops much more slowly in the spontaneous models than it does in induced and post traumatic models. In addition, the severity of the disease and speed of onset may vary widely between subjects in naturally occurring OA [10]. Furthermore, it is not certain that each mouse will develop OA [10]. The incidence of OA in wild type mice is estimated to be 20–45% [11]. This means that many mice need to be raised for an extended period of time and most of the mice raised will not be viable test subjects because they do not end up developing OA [10, 11]. The uncertainty makes this method time-consuming, inefficient, and expensive compared to other methods. One way to solve the inefficiencies of the spontaneous model is to use genetically modified models [10, 11].

### Genetically modified models

In certain other cases, researchers select mice with genetic modifications that make the development of OA more likely. These models are also “spontaneous” as there is no intervention applied that directly causes OA, but these mice have a much higher incidence of OA than their wild type counterparts. For example, Lapvetelainen et al. found that the incidence of OA in mice with knockouts of the Col2a1 gene was 60–90%, which is much higher than the incidence in wild type mice [11]. Another example of a commonly used strain of mice is STR/ort mice [5]. These mice have certain characteristics which make them more likely to develop OA. For instance, they have increased levels of inflammatory cytokines including IL1β, IL12p70, MIP1β, and IL5 [12]. These changes are associated with worsened bone morphology and the development of OA. These mice commonly develop OA in knee, ankle, elbow, and temporo-mandibular joints [13]. Kumagai et al. found the incidence of OA in STR/ort mice to be 68% [14]. The incidence of OA is higher in male STR/ort mice than in their female counterparts; this is the opposite of the incidence observed in humans, where females are more likely to develop the disease [15]. These mice generally develop OA at around 18 weeks of age. Once OA develops, it progresses rapidly and becomes more and more severe. Staines et al. note in their description of the STR/ort model that one unique aspect of this model is that it highlights the genetic factors that impact the development and health of articular chondrocytes and subchondral bone, making it particularly useful in studying the progression of OA [16]. The STR/ort mouse model was used in studies that have deepened our understanding of the pathophysiology of OA, including studies which demonstrated the role of chondrocyte metabolism in the progression of OA [5, 12].

Pasold et al. used this model in a study in which they investigated genes associated with OA. They found that in STR/ort mice, there is a reduced expression of the gene Sfrp1. This is associated with increases in b-catenin and decreases in wnt signaling. Both of these factors render articular chondrocytes more susceptible to premature aging and damage resulting in OA. These genes could be potential targets for therapeutic agents for OA [17].

Another example of a genetically modified model is the use of Col9a1(−/−) mice [18, 19]. These mice have collagen type IX alpha 1 gene inactivation and they are frequently used to characterize the role of collagen type IX in the pathogenesis of OA [18, 19]. Costello et al. used this method and found that male mice homozygous for this gene inactivation developed OA and associated increased tactile pain sensitivity and gait alterations by 9 months of age [20]. They were able to use this model to evaluate the degree to which OA impacts locomotor activity in mice [20]. Another genotype of mice that is highly susceptible to OA is interleukin-6 knockout gene mice (IL-6(−/−)) [21]. These mice have a deficiency in interleukin 6 which results in decreased proteoglycan synthesis and reduced bone morphology density among male IL-6(−/−) mice compared to their wild type counterparts. This leads to the development of more severe OA among these mice [21].

On the other hand, mice can also be genetically modified in an attempt to make them less susceptible to OA. For instance, mice with a knockout gene that encodes a protease specific to the knee joint might be less likely to develop OA than wild type mice. Genetically modifying mice to find protective factors is a promising avenue in developing future treatments for OA [22]. For example, Motomura et al. found that c-Fos/activator protein (AP)-1 inhibitor, T-5224, which inhibits matrix metalloproteinases (MMPs) prevents cartilage destruction and reduces rates of OA in mice [22]. The advantage of using mice which are likely to develop OA is that it saves researchers time and money compared to using wild type mice which have a lower incidence of OA [5, 6, 8]. Due to the tremendous variability in susceptibility to OA among different strains of mice, it is very important that any study using mice specify the type of mice used in the study [23].

## Induced models of osteoarthritis

### Surgically induced models

One common method for inducing OA in mice is surgical induction of OA. In this method, a surgeon performs an operation to induce an injury which will eventually lead to the development of OA in the desired joint [5, 10]. The most commonly used surgical method of inducing OA is anterior cruciate ligament transection (ACLT) [9, 10]. In this method, a surgeon transects the subject’s ACL, which causes joint destabilization. The anterior drawer test with the joint flexed is used to confirm that transection of the ligament has occurred. In some cases, other ligaments such as the posterior cruciate ligament, medial collateral ligament, lateral collateral ligament, and/or either meniscus may be transected as well [9, 10]. Transection of different combinations of the ligaments of the knee allows researchers to examine the progression of osteoarthritis as it relates to a wide variety of different types of trauma and degeneration. Eventually, this joint destabilization induces post traumatic osteoarthritis (PTOA) in the knee joint which was subjected to the ACLT. This mimics the development of post traumatic OA in human beings, which follows a similar pattern of pathogenesis [9, 10]. Recently, Zhen et al. used an ACL Transection OA model in a study in which they describe the role of transforming growth factor-beta (TGF-β) in osteoarthritis. They found that TGF-β contributes to the degradation of subchondral bone and that inhibition of TGF-β via TβRI inhibitor had the therapeutic effect of reducing articular cartilage degradation in mice with OA [24]. ACL Transection is the most commonly used method of inducing osteoarthritis [6, 10]. There are numerous benefits to this method. The ability to quickly and reliably induce OA as well as the repeatability of the induction are two of the most commonly cited advantages for the ACLT method.

Another commonly used surgical method for inducing OA is a meniscectomy. In this method, the medial collateral ligaments are cut in order to expose the meniscus. Then, the surgeon severs the meniscus to destabilize the joint [25]. Either the lateral or medial meniscus can be chosen for transection. In mice, it is more common to sever the medial meniscus as it generally bears a heavier load and thus is more likely to lead to the development of OA when it is severed [25]. When the medial meniscus is severed, this method is often referred to as destabilization of the medial meniscus or DMM [25]. This method produces rapidly progressing PTOA with characteristics that mirror the progression of the disease in humans. In humans, 50% of people who undergo a meniscectomy develop OA within 20 years of the date of the surgery [25]. In mice, mild osteoarthritic defects begin to develop rapidly after the procedure is performed. The OA tends to progress from mild to moderate at 4 weeks after the date of the surgery and from moderate to severe OA at 8 weeks after the surgery [26]. Jia et al. utilized this method in a study in which they investigated the mechanisms underlying subchondral bone plate (SBP) sclerosis in mice with late state OA. They found SBP sclerosis primarily in areas underneath severely eroded articular cartilage. They also found a decrease in the levels of sclerostin in the SBP of mice with DMM induced OA [27]. Sclerostin is a small protein expressed by chondrocytes and osteocytes; it is not completely understood how reduced levels of sclerostin contribute to osteoarthritis but it is hypothesized that sclerostin prevents Wnt-pathway modulated expression of disintegrin and metalloproteinase [27].

A final surgical method of inducing OA is ovariectomy. In this method, the ovaries are surgically removed. The rationale behind this method is that estrogen serves as a protective factor against osteoporosis and OA. This is why the incidence of osteoporosis and OA is much higher in post-menopausal women [28]. Removing the ovaries reduces estrogen levels and mimics the physiological changes that occur in menopause. Therefore, this method could be useful for studying the pathophysiology of OA. It also has utility in studying therapeutic methods for alleviating pain and slowing the progression of the disease [28]. However, there is some debate as to the efficacy of this method. In a review of the effect of ovariectomy on different animals, it was found that the literature is inconclusive on whether ovariectomy reliably induces OA on its own. Ma et al. found that ovariectomy did increase the incidence of OA compared to shams; this effect was greater when used in conjunction with another method [29]. On the other hand, Chambers et al. found that mice who were subjected to ovariectomy were no more likely to develop OA than their counterparts who were subjected to a sham procedure [30]. The efficacy of this method in its own is not clear. It has been shown to reliably cause more severe OA when used in conjunction with another method of inducing OA [28–30].

The surgical method for inducing OA also has a number of drawbacks. One major drawback is that a skilled surgeon is needed to perform the procedures in a reliable and consistent way across all subjects [10]. If there are inconsistencies in the way the procedure is performed on different subjects, it could confound the data and make it impossible to draw meaningful conclusions from the study. Another drawback of the surgical method is that anytime surgery is performed, there is the possibility of infection [5, 10]. Developing an infection during surgery could dramatically alter the progression of OA in the test subject. Therefore, it is vitally important for researchers using the surgical method to be vigilant in preventing infections. Another drawback of this method is that the disease develops very rapidly following the surgically induced trauma. This can be useful for researchers who want to study the late stages of the disease; however, those who are interested in the early stages of OA will not be able to study the disease using this method as the onset is too rapid [5, 6, 10].

### Chemically induced models

Another common type of model for inducing OA in mice is chemically induced models. In these methods, an inflammatory or toxic compound is injected directly into the joint in which OA is to be induced. These compounds interact with the structures that comprise the joint and compromise their function in different ways in order to induce OA [29–31]. In the past, the use of papain to induce OA was common. Papain is a proteolytic enzyme which causes degeneration when injected into a joint. The use of papain is becoming less common as other, more effective chemical models of inducing OA have been developed [31]. One of the chemically induced models that has begun to be used more frequently is the injection of collagenase into the joint [32, 33]. Intra-articular administration of collagenase leads to the breakdown of the collagen fibers (Type I) within the cartilage. This leads to a reduction of the collagen matrix in the tendons and ligaments, which consequently causes joint instability [32, 33]. Collagenase injections are generally performed twice; each injection is a dose of either 250 U or 500 U and injected into the joint through the patellar ligament via a 26 G needle. The second injection is typically applied 3–5 days after the first injection [30, 31]. The collagenase is dissolved in saline for the injection]. The injection of collagenase was found to induce changes that closely mimic human OA. The joint instability that develops over time makes this an effective method to study pain behavior in the subject that arises in response to gradual changes in the function of the injected joint [33, 34].

Recently, Cosenza et al. utilized the collagenase induced model in a study published in 2017 in which they found that bone marrow derived mesenchymal stem cells (MSC) had a protective effect on the chondrocytes of mice with collagenase induced OA. They found that the MSCs were effective in re-establishing chondrocyte homeostasis and relieving inflammation in the joint. This shows how the collagenase induced model was able to be used effectively in a study evaluating possible therapeutic agents for OA [35].

Mono-iodoacetate (MIA) is another example of a compound that can be injected into the joint of a mouse to induce the development of OA. In this method, a solution of MIA in saline is injected into the hind knee of an anesthetized mouse [36]. The knee is held in a bent position and then a 26G needle is used to inject the solution just below the patella. This leads to the inhibition of glyceraldehyde-3-phostphate dehydrogenase, causing an interruption of the Krebs cycle which leads to chondrocyte death [36]. Following chondrocyte death, osteophytes form and articular cartilage is slowly degraded. Inflammation and pain begins quickly and persists for at least 7 days following injection. Chronic musculoskeletal pain generally develops within 10 days of the date of injection [33, 35]. This model is effective in causing the development of OA; however, there is some debate as to how effective it is as a model of naturally occurring OA. MIA is a metabolic poison and causes widespread chondrocyte death. The pathophysiology of the osteoarthritis induced this way is slightly different than the way the disease progresses in humans [23]. Therefore, this model is not commonly used to study the progression of OA as the damage it causes does not mimic true OA as well as certain other models. Instead, it is mainly used to observe and evaluate pain behavior and to assess the efficacy of therapeutic interventions to reduce inflammation and pain [36–39].

Guingamp et al. found dose dependent reductions in mobility in response to injection of MIA [39]. High doses (0.3 mg and 3.0 mg) led to changes in cartilage proteoglycan concentration and inhibition of patellar anabolism 15 days after injection. They concluded that injection of high doses of MIA is a quick and effective way to induce OA in mice [39]. Pitcher et al. conducted a study in which they evaluated the utility of this model as a method for measuring mechanical hypersensitivity (allodynia) and weight bearing deficits as a result of OA [36]. They found that injection of 0.5–1.0 mg of MIA in the knee joint of a mouse leads to ipsilateral mechanical hypersensitivity of the hind paw and decreased weight bearing ability for 4 weeks following the injection [36]. Pitcher et al. suggested this as an effective method for studying pain behavior in response to OA [36]. One limitation of this method mentioned by Pitcher et al. is that the rapid onset does not accurately simulate the slow, progressive onset of OA in human beings. They suggest that this limitation could be overcome by using the MIA injection model in conjunction with a surgical model of inducing OA [36]. Both of these studies illustrate the efficacy of the MIA chemically induced model of OA [36, 39].

One drawback ubiquitous to chemically induced models is that they have a different pathophysiology than post-traumatic OA. This means that they are not effective in examining the usual progression of the disease in humans. However, they do have utility for studying the mechanism by which OA causes pain and the behavioral changes resulting from this pain. Also, these models are useful for evaluating the efficacy of different drug therapies which can be used to alleviate the pain experienced by people afflicted with OA [32–39].

### Non-invasive induced models

Another category of models of OA is non-invasive post traumatic models. These models rely on automated application of trauma to the joint to induce injuries similar to those induced in the surgical models [40]. These models were developed to eliminate some of the confounding factors in the more invasive surgical and chemical methods of inducing OA. Since there is no surgery required, there is no chance of infections, which are a major confounding factor in studies that utilize surgical models of inducing OA [40]. A major drawback of the surgically induced models of OA is that it is impossible to perfectly replicate surgical trauma across test subjects. This introduces a degree of variability that could be an experimental confound. In non-invasive models, the trauma is applied automatically; therefore, the force applied is exactly the same for all subjects creating consistent levels of trauma and similar progression of OA. This eliminates the variability of the surgical models and makes it easier to draw statistically significant conclusions from studies [40]. The three most common non-invasive methods of inducing OA are described below:

1. Intra-articular tibial plateau fracture: This was the first non-invasive method developed. In this method, the flexed knee of an anesthetized mouse is fixed on a triangular cradle while an indenter provides the force of impact [40, 41]. One study that describes this model used a 10 N compressive preload followed by a compressive load of 55 N delivered at a rate of 20 N/s. This load was found to be sufficient in force to cause a fracture and subsequently induce the development of OA as shown by histological staining and evaluation with microCT [42]. This method can be used to replicate acute trauma in human beings including injuries from high energy impacts such as motor vehicle accidents [42]. Traumatic injuries are a common cause of OA; some studies have estimated the incidence of PTOA following significant joint trauma to be as high as 75% [43]. Articular fractures in particular have been estimated to increase the risk of developing OA as much as 20 fold [43, 45]. This model allows researchers to quickly and consistently cause an injury that causes many cases of OA in human beings [43, 44]. This method is often used to study the pathophysiological changes that occur in early stages of the progression of OA. One major advantage of this method is that the severity of the lesions can be adjusted to simulate varying degrees of traumatic injuries [5]. One major drawback of this method is that the results of this model are not applicable to cases of OA caused by overuse and chronic injuries [5, 42]. Lewis et al. utilized this method in a study in which they studied the degree to which fracture severity impacts the joints. They found significant decreases in chondrocyte viability as well as increases in biomarkers associated with OA in mice subjected to more severe tibial plateau fractures [45]. Furman et al. used this type of model in a study in which they attempted to inhibit the inflammatory response that contributes to the progression of OA. They found that intra-articular inhibition of interleukin 1 (IL-1) reduced inflammation and degradation of cartilage following an articular fracture of the knee [46]. They concluded that in mice, injection of IL-1 inhibitor IL-1Ra effectively reduces arthritic damage [46]. However, these results have not been replicated in human clinical trials.
2. Cyclic articular cartilage tibial compression (CACTC): In this method, an axial load is applied to displace the tibia relative to the femur [40, 42]. The load can be applied in cycles over time or as a single one-time overload if the goal is to induce an ACL rupture [47]. The second possibility will be discussed in greater detail in the next paragraph. Most commonly, the load is applied three times a week for 2–5 weeks to ensure the best results [48]. This model is also more effective when the loading regimen is followed by 2 weeks of non-loading, during which time the mice resume normal cage activities [47, 48]. The constant use of the injured joint leads to further damage which closely mimics the progression of osteoarthritic joint degradation in human beings [48]. This method is the preferred method to study the effect of chronic overuse injury on the development of OA [48]. One drawback of this method is that it is not useful for acute injuries. Another drawback of the CACTC model of OA is that several cycles of tibial compression must be applied and it takes longer for the test subject to develop OA in this model than in other induced models [48].
3. Anterior cruciate ligament (ACL) rupture via tibial compression overload: In this method, Anterior subluxation of the tibia is used to produce injury. A 12 N load is applied to the knee to cause the injury. The load can be applied either slowly (1 mm/s loading rate) or more quickly (speeds as as high as 500 mm/s loading rate). The injury pathology and development of OA are similar to the animal ACL transection model but without the need for invasive surgery [49]. In a study evaluating the efficacy of this method, Christiansen et al. found degradation of trabecular bone within 7 days of the injury and osteoarthritic changes within 56 days [44]. Lockwood et al. conducted a study in which they compared the slow loading and fast loading models. These two models showed significant osteoarthritic damage 12 and 16 weeks post injury respectively [50]. Khorasani et al. used this method in a study in which they evaluated alendronate (ALN) as a potential therapeutic agent for OA. They used ACL rupture via tibial compression overload to induce osteoarthritis in 90 mice. The mice were then injected twice weekly with ALN (40 μg/kg/dose) or high-dose ALN (1000 μg/kg/dose). MicroCT was used to evaluate cartilage damage. They found that alendronate is effective in preventing early bone loss after injury but not effective in preventing long term cartilage degradation [51]. One major advantage of this method is that the traumatic load must only be applied a single time to produce the desired results. Rai et al. utilized this method to study the pathophysiology of OA in mice and found the progression of the disease to be similar to, albeit faster than, the progression of the disease in humans [52]. Based on the results of their study, they identified chondrocyte apoptosis, synovitis, and ectopic calcification as possible targets for therapeutic interventions [52].

All of these models eliminate the most pressing issues with the surgical methods of inducing OA. They eliminate the possibility of infections and do not require a trained surgeon [5]. However, these methods have their own drawbacks. One major drawback of the non-invasively induced OA models is that they are relatively new and therefore there is minimal literature on their application and efficacy [5]. Furthermore, the equipment needed to automate the application of traumatic forces is expensive, which can be cost prohibitive in cases when researchers are working with a limited budget. For labs that will not use the equipment often, the equipment may not be worth the hefty cost.

## Limitations of murine OA models and use of other animal models

While murine models of osteoarthritis are versatile and enable us to learn a great deal about the pathophysiology of osteoarthritis, it is important to keep certain limitations in mind that pertain to all different models. There are limitations ubiquitous in all animal models including differences in size and gait [5]. Furthermore, there are certain limitations specific to mice. A lack of intra-cortical bone remodeling during loading periods as well as differences in the articular cartilage between humans and mice make it impossible to be sure whether findings in murine models will be applicable to human beings. However, on the whole these models have proven useful in studying OA [5, 6, 10].

Other animals including horses, guinea pigs, rats, pigs, and sheep are also used to study OA. The horses and goats are two of the most frequently used large animal models whereas guinea pigs are one of the most commonly used small animal models after mice.

Horse models of osteoarthritis are considered to be particularly useful because the articular cartilage of horses and subchondral bone thickness are the closest to those of humans out of any of the animals commonly used in research [54]. Horse models have several other advantages. The large size of the joint allows OA to be induced via arthroscopic surgery. Further arthroscopies can be performed at a later time to observe changes over time in the joint [54]. The most commonly used model to induce OA in horses is arthroscopically created osteochondral fragment-exercise model. This model, which was developed by McIlwraith et al. is considered to be better than other horse models of OA because it induces progressive OA without creating instability, which is common in other models and often causes the horse to become lame [54].

Other models used in horses include the injection of chemicals into the joint, induction of instability via trauma, and repeated overloading can all be used to induce osteoarthritis in horses [54]. Many of these models are very similar to models used in mice. Carrageegan can be injected into horse joints to promote inflammation and induce osteoarthritis [5]. In addition, mono-iodoacetate can be effective in inducing OA in horses just as it is in mice. Elmesirty et al. found that injection of 50 mg of mono-iodoacetate into the joint leads to synovitis within a week which subsequently progresses to osteoarthritis within 70 days [55].

Trauma can be used to induce osteoarthritis in the medial femorotibial joint of horses. Bolam et al. characterized this method. A contusive impact is applied to the medial femoral condyle of horses and leads to the development of osteoarthritis within 56 days [56]. Repeated overloading is another way in which osteoarthritis can be induced in horses. Turley et al. described pathological changes in the joints of race horses as a result of years of training. Many of these horses had osteoarthritis as a result of constant heavy use of the joints. They found that these horses were useful models for studying the histo-pathology of OA and for observing pain behavior in response to the progression of OA [57]. Since horses are more anatomically similar to humans than many other animals, they are often used to confirm the efficacy of drugs and other therapeutic strategies before beginning clinical trials in humans.

Sheep are another species large animal that can be used to study osteoarthritis. Sheep are particularly useful in studying osteoarthritis of the knee because the knee is very similar anatomically to the human knee [58]. Furthermore, the large size of the joint relative to mice and guinea pigs makes procedures such as ACL transection easier to perform in a consistent way. Surgical induction is by far the most commonly used method for the induction of osteoarthritis in sheep. ACL Transection and Medial Meniscectomy are the two most common methods for achieving this [59]. The procedures are performed in a very similar way to the way they are performed in mice; osteoarthritis tended to develop within 12 weeks of the surgery [59–62]. It is also possible to utilize partial meniscectomies to induce osteoarthritis in sheep. Cake et al. outline some of these methods. They compared total meniscectomy with mid-body transection, in which only a portion of the meniscus is transected, and cranial pole meniscal release, in which the cranial meniscotibial ligament is isolated and transected. They found that all three procedures were equally effective in inducing osteoarthritis in sheep.

Ovine (sheep) models are particularly useful in evaluating different therapies for osteoarthritis. Spadari et al. used the meniscectomy method to evaluate the efficacy of stanozolol as a therapeutic agent [60]. They found that intra-articular injection of stanozolol reduced osteophyte formation and promoted articular cartilage regeneration [60]. Delling et al. used a lateral meniscectomy method to induce osteoarthritis in sheep. They evaluated the efficacy of mesenchymal stromal cell administration on osteoarthritis in sheep. They found that there was no significant benefit in the severity of osteoarthritis compared to a control group [59]. Song et al. performed a study in which they evaluated bine marrow mononuclear cells and bone mesenchymal stem cells as possible therapeutic agents. They utilized both meniscectomy and ACL transection to induce osteoarthritis in their subjects. They found that both bone marrow mononuclear cells and bone mesenchymal stem cells have similar therapeutic potential but that the bone marrow mononuclear cells are easier to procure and isolate and thus may be a better therapeutic agent [59]. These studies illustrate the fact that the ovine model is preferred for the evaluation of potential therapeutic agents for osteoarthritis. The degree of similarity between human joints and those of sheep makes these models useful in determining which interventions might be suitable for testing on humans. Surgical induction is most commonly used for this purpose because the pathophysiology is closest to that of naturally-occurring osteoarthritis.

Guinea pigs are another small animal that is used as a model for OA. The Dunkin Hartley Guinea Pig model is the most commonly used guinea pig model for studying osteoarthritis [63]. Kim et al. characterized this model and found that the progression of OA is Dunkin Hartley guinea pigs developed naturally occurring, time-dependent osteoarthritis that closely mimicked the course of the disease in humans [63]. They found the naturally occurring Dunkin Hartley guinea pig to be among the best models for studying the pathophysiology of naturally occurring OA. Tonge et al. studied the development of OA in Dunkin Hartley guinea pigs. They found that OA started to develop when the guinea pigs were between 2 and 3 months old and developed over time until the guinea pigs were around 7 months old [64]. They were able to draw some important conclusions about the pathophysiology of OA. They found that the onset of OA coincided very closely with increased expression of MHC IIX mRNA [64]. They believe that this mRNA affects the fast-twitch muscle fibers of the quadriceps, and that changes in the contractile properties of these fibers contribute to the development of OA [64]. This example shows the utility that naturally occurring OA in Dunking Hartley guinea pigs has in studying the development of OA.

Kim el al also utilized a chemically induced model of OA in guinea pigs; they injected mono-iodoacetate into the joints of guinea pigs and found that this was a simple and reliable way to induce OA in guinea pigs [63]. The mechanism by which mono-iodoacettate causes OA in guinea pigs is similar to the way it does so in mice; it acts as a metabolic poison and causes widespread chondrocyte death. This means that guinea pigs with mono-iodoacetae induced OA are useful studying pain behavior in response to OA but not the progression of the disease, nor potential treatments. Finally, OA can also be induced in guinea pigs via surgical methods, for instance meniscectomy [8]. Guinea pigs are a good choice for medial meniscitomy studies because both guinea pigs and human beings tend to load the medial meniscus more than the lateral meniscus, so destabilizing the medial meniscus will lead to a similar disease progression [8].

Overall, the naturally occurring Dunkin Hartley model is by far the most utilized guinea pig model of OA. This model has great utility in studying the pathophysiology of OA. For most other purposes, mice are preferred to guinea pigs because they are cheaper to raise and develop OA quicker than guinea pigs do [63, 64]. There are many animal models apart from mice that may be more applicable depending on the aims of the study. Table 3 summarizes the primary uses of each of the different animal models that were discussed in this section.

### Concluding remarks

This study examines some of the plethora of methods for inducing OA in mice for the purpose of studying the progression and pathophysiology of the disease, as well as for evaluating the efficacy of possible therapeutic agents. All of the models discussed in this paper have been shown to be effective in inducing OA in mice. However, each model has advantages and disadvantages which must be taken into account when deciding which one is the best to use for a specific study. There is not one single best model of inducing OA; one must select the model that is most applicable to the study one is conducting. The purpose of the study (whether the focus is on studying the pathophysiology and progression of the disease or evaluating therapeutic agents), budget, availability of equipment and surgeon, and time frame must all be considered in order to choose the method that will be most effective and applicable. We have summarized the different models and the types of studies for which they are useful in Table 2.

**Table 2:** This table indicates the appropriate uses for each type of murine OA model. An X indicates that a model is appropriate for a specific type of study

	OA pathophysiology	OA progression	OA therapies	Pain behavior	Post-traumatic OA studies	References
Naturally occurring	X	X	X	X		[9–11]
Genetically modified	X	X	X	X		[12–24]
Surgically induced (all)	X	X	X	X	X	[25–30]
Chemically induced (all)				X		[31–39]
Non-invasive induction	X	X	X	X	X	[40–52]

For studies that aim to study the pathophysiology of osteoarthritis, the naturally occurring models, including those with genetic modifications, are ideal. They give the closest representation to the progression of primary OA in humans and have little risk of infection or confounding factors related to induction. However, these methods are time consuming and inefficient, and thus their use may not always be feasible. Furthermore, there is some degree of variability between the subjects. The surgically induced and non-invasive models are more reliable and have more consistency across all subjects in the study Researchers who need a large number of subjects with similar degrees of OA may be inclined to select a surgically induced or non-invasive model.

Genetically modified models are particularly useful to determine the impact of genetics on OA development and how different people might respond differently to specific interventions. For studies on pain behavior in response to OA, chemically induced models are ideal. For studies that hope to test the efficacy of a medication or therapeutic intervention, most models except for the chemically induced models are appropriate. The ideal model for these studies depends on the type of OA that the intervention aims to treat. The spontaneous models are best for evaluating treatments that are specific for primary OA. On the other hand, the surgically induced and non-invasive models are better for evaluating treatments for post-traumatic OA. Studies on possible therapeutic interventions should first be conducted on small animals such as mice or guinea pigs, and then move on to larger animals, such as horses or sheep, which are anatomically similar to humans and therefore likely to more effectively predict the effect the intervention will have in humans. Any induced model is useful for comparing one joint against another within an animal because OA can be induced in only one joint whereas spontaneous OA is likely do develop in multiple joins at once due to the progressive nature of the disease. Based on our understanding of the current literature regarding animal models of OA, we have summarized the most commonly utilized animals and models in Table 3.

**Table 3:** This table summarizes the most frequently used models for each of the animals discussed in this review

Animal	Frequently used models	References
Mouse	Spontaneous Chemically induced Surgically induced Non-invasive induction	[5, 10–52]
Horse	Osteochondral fragment-exercise model Chemically induced Induction via trauma Induction via overuse	[5, 54–57]
Sheep	Surgically induced	[5, 58–62]
Guinea Pig	Spontaneous (Dunkin Hartley) Chemically induced Surgically induced	[5, 8, 63, 64]

## Abbreviations

OA: osteoarthritis
IL: interleukin
MMP: matrix metalloproteinase
ACL: anterior cruciate ligament
ACLT: anterior cruciate ligament transection
PTOA: post traumatic osteoarthritis
TGF-β: transforming growth factor-β
DMM: destabilization of the medial meniscus
SBP: subchondral bone plate
MSC: mesenchymal stem cell
MIA: mono-iodoacetate
CACTC: cyclic articular cartilage tibial compression
ALN: alendronate
